# Exploring the Impact of Olive-Derived Bioactive Components on Gut Microbiota: Implications for Digestive Health

**DOI:** 10.3390/foods14142413

**Published:** 2025-07-08

**Authors:** Manuel Garrido-Romero, Marina Díez-Municio, Francisco Javier Moreno

**Affiliations:** 1 Instituto de Investigación en Ciencias de la Alimentación, CIAL (CSIC-UAM), Nicolás Cabrera 9, 28049 Madrid, Spain; manuel.garrido@pharmactive.eu; 2 Pharmactive Biotech Products SLU, Faraday 7, 28049 Madrid, Spain; mdiez@pharmactive.eu

**Keywords:** olive, bioactive compounds, gut microbiota, Mediterranean diet

## Abstract

Recent advances highlight the crucial role of the gut microbiota in human health and disease, with dietary components emerging as powerful modulators of microbial communities. This review synthesizes current evidence on the effects of olive-derived bioactive compounds, including polyphenols (e.g., hydroxytyrosol, oleuropein or tyrosol), triterpenes and other phytochemicals on gut microbiota composition and function. These compounds have been shown to enhance beneficial bacterial populations such as *Lactobacillus* and *Bifidobacterium*, reduce potentially pathogenic taxa, and promote the production of short-chain fatty acids and other health microbial metabolites, reinforcing intestinal barrier integrity. In vitro, in vivo, and clinical studies also reveal the potential of olive bioactives to ameliorate metabolic, inflammatory, and neurocognitive disorders through gut-microbiota-brain axis modulation. Despite promising results, key challenges remain, including interindividual microbiota variability, lack of standardized intervention protocols, and limited human clinical trials. Addressing these gaps through robust translational research could pave the way for microbiota-targeted, personalized nutritional strategies based on olive-derived compounds.

## 1. Introduction

Over the past decade, the intricate relationship between diet and the gut microbiota has become one of the most vibrant areas of biomedical and nutraceutical research. Once viewed simply as a passive community of commensals, the trillions of microorganisms inhabiting the human gastrointestinal tract are now recognized as an active “organ” that influences nutrient metabolism, immune maturation, and even the gut–brain axis [1]. Dietary components not only may serve as substrates for microbial growth but also exert selective pressures that shape community composition, microbial gene expression and the generation of bioactive metabolites such as short-chain fatty acids (SCFAs), secondary bile acids, and phenolic derivatives [2].

As such, food is increasingly valued not only for its macronutrient and micronutrient content but also for its capacity to modulate microbial ecosystems and, by extension, host physiology. Within this framework arises the concept of functional foods, defined as those that, beyond basic nutritional value, contain components capable of promoting health or reducing disease risk. Such components include soluble and insoluble fibers that function as prebiotics, SCFAs, bioactive peptides, and other natural compounds that interact with both the microbiota and the host [3]. Studying these food ingredients and their mechanisms of action is key to designing dietary strategies aimed at preventing digestive disorders and improving overall well-being.

A well-established example of a dietary pattern rich in functional component is the Mediterranean diet, long associated with reduced risk of cardiovascular and metabolic diseases. It is distinguished by its high intake of fruits, vegetables, legumes and nuts, moderate consumption of fish and wine, and, in particular, its liberal use of extra-virgin olive oil [4]. This dietary pattern famously places olives and their derived products, especially extra virgin olive oil, at the very base of its traditional nutrition pyramid [5].

Beyond the olive oil itself, other derivatives such as olive leaves, pomace and mill waste water represent abundant reservoirs of these same metabolites, offering opportunities for sustainable valorization of by-products [6]. These olive by-products are highly valued across a range of industries, including the nutraceutical, cosmetic, pharmaceutical, and bioenergy sectors [7].

**Figure 1:**
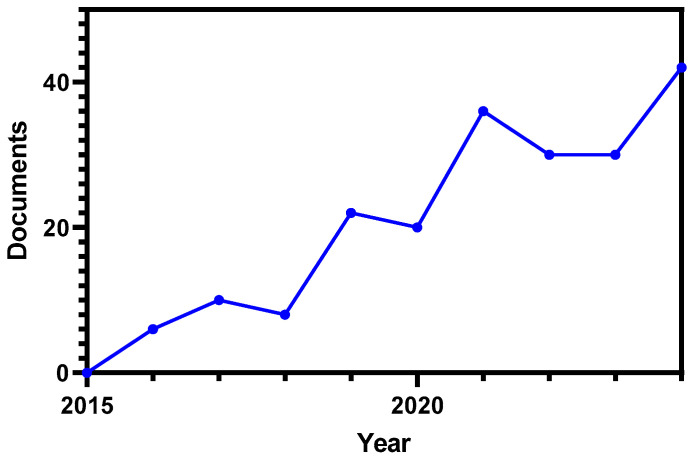
Progression of scientific production regarding olive and gut microbiota (2015–2024). The data was retrieved from Scopus using the search query: TITLE-ABS-KEY ((“Olive” or “Olea”) AND “Gut microbiota”).

Despite the increasing number of studies or reviews on this topic (Figure 1), there is currently no comprehensive synthesis that brings together the diverse findings on olive-derived compounds and their impact on the gut microbiota. A critical and integrative review is thus warranted to evaluate the consistency of the available evidence, identify gaps in knowledge, and inform future directions in both scientific research and the development of novel functional foods. This review aims to address these needs by systematically examining the interactions between olive bioactive compounds and the gut microbiota, with a particular focus on underlying mechanisms, therapeutic potential, and innovation opportunities.

## 2. Olive and Its Main Bioactive Compounds

The olive tree *(Olea europaea)* is a Mediterranean native plant widely cultivated for its fruit and oil, both of which have been valued for centuries due to their nutritional and medicinal properties. Originating from the eastern Mediterranean basin, olives are now widely grown in Southern Europe, North Africa, the Middle East, and parts of the Americas and Australia. Among the most common cultivars are ‘Arbequina’, ‘Picual’, ‘Koroneiki’, and ‘Frantoio’, each differing in their phenolic profile, oil yield, and resistance to stress [8].

Various parts of the olive tree are recognized for their nutritional value, functional ingredients, and bioactive compounds that contribute to health promotion. The olive fruit is particularly notable for its abundance of phenolic compounds, including oleuropein, hydroxytyrosol and verbascoside, as well as dietary fiber and minor amounts of triterpenes [9]. Olive oil, extracted from the fruit pulp, is a major source of monounsaturated fatty acids, especially oleic acid, and also provides tocopherols, phytosterols, squalene and polyphenols, all of which have been linked to numerous health benefits [10]. While olive oil contains the lipophilic forms, other by-products often hold a higher concentration of the more hydrophilic or glycosylated phenolic forms:

- **Olive leaves**: they are a by-product of olive cultivation and pruning, and are rich in secoiridioids such as oleuropein and its derivatives, along with flavonoids. These compounds exhibit antioxidant and anti-inflammatory properties and are being explored for use in functional foods and gut microbiota modulation [11,12].
- **Olive pomace**: is the solid residue from oil extraction and is a particularly rich source that contains not only residual oil but also substantial amounts of dietary fiber, lignans, and a wide spectrum of phenolic compounds that often mirror those found in the oil but may include unique derivatives [13].
- **Olive mill wastewater**: is a notable reservoir of water-soluble phenolics, including hydroxytyrosol. Despite being traditionally considered an agro-industrial by-product, it has attracted growing interest for its antioxidant, antimicrobial, and anti-inflammatory potential, making it a valuable source for the recovery of functional ingredients in the context of circular economy and sustainable bioproduct development [14].

### 2.1. Understanding Olive’s Bioactive Components

Olive is an abundant source of bioactive compounds with significant health potential, whichare found across different parts of the plant [15]. Among them, phenolic compounds, a diverse group of secondary metabolites that includes polyphenols, phenolic acids, flavonoids, and other structurally related compounds, are the most extensively studied due to their broad range of biological activities, including antioxidant, anti-inflammatory, antimicrobial, and microbiota-modulating effects [16,17], positioning them as valuable agents for use in functional foods, nutraceuticals, and therapeutic applications [18].

Olive contains over 170 identified polyphenols, compounds known for their strong antioxidant capacity, which helps to reduce oxidative stress and inflammation by neutralizing reactive oxygen species and influencing expression and promoting beneficial epigenetic changes [19]. They also exhibit prebiotic-like effects, shaping gut microbiota and enhancing the bioactivity of phenolics through microbial metabolism by producing SCFAs [20,21]. Additionally, polyphenols can regulate the pathways that control lipid metabolism and glucose homeostasis, which are crucial for preventing metabolic disorders such as obesity and type 2 diabetes [22].

Among polyphenols, flavonoids are especially notable for their antioxidant, anti-inflammatory, and anticancer properties, primarily due to their capacity to neutralize free radicals and modulate key cellular signalling pathways such as NF-κB and MAPK [19,23]. These compounds also have the ability to influence the activity of detoxifying enzymes and inhibit pro-inflammatory mediators, contributing to their therapeutic potential. In olive-derived products, flavonoids are particularly abundant in leaves and to a lesser extent in fruits and oil, and the main flavonoids identified include luteolin-7-glucoside, luteolin-5-glucoside, apigenin-7-glucoside, apigenin-7-rutinoside, and quercetin-3-rutinoside [24].

**Figure 2:**
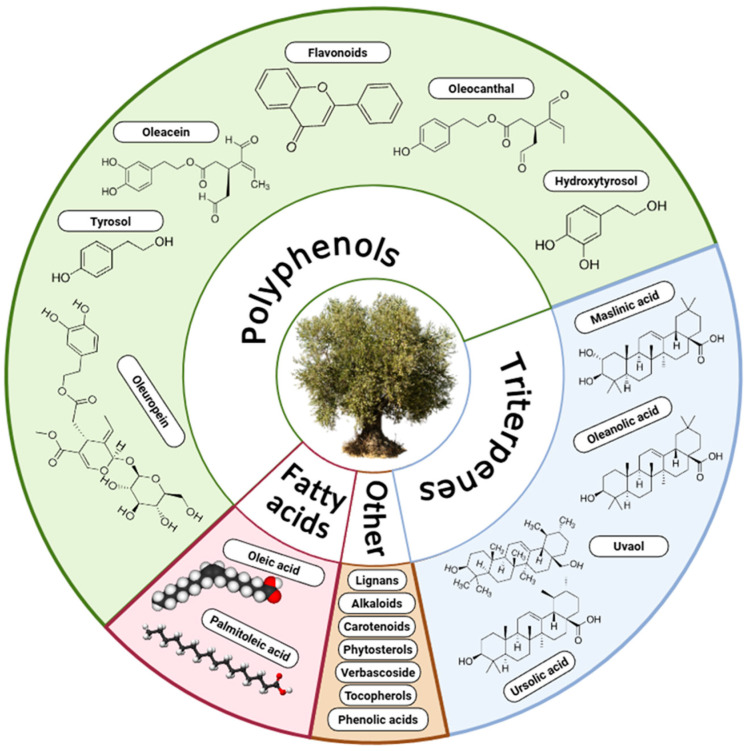
Main bioactive compounds in olive.

Olives are particularly rich in distinctive phenolic compounds, especially secoiridoid derivatives, which together define much of the olive’s bioactive potential: (Figure 2):

- **Oleuropein**: is a molecule characterized by a hydroxyphenyl group attached to a secoiridoid aglycone. It is known to exert a broad spectrum of beneficial effects, including anti-inflammatory, anticancer, antimicrobial, and neuroprotective actions [25]. Additionally, oleuropein has been shown to modulate beneficial skin-associated [26] and gut microbiota [27].
- **Hydroxytyrosol**: is a phenylethanoid compound that is well known for its strong antioxidant capacity, which is largely attributed to its catechol structure bearing hydroxyls groups at the 3- and 4- positions, functional groups that play a key role in neutralizing free radicals [28]. In addition, hydroxytyrosol demonstrates an exceptional bioavailability of 99% [12], ensuring efficient absorption and systemic distribution within the human body and in 2012 the EFSA approved its role in the prevention of atherosclerosis [29].
- **Tyrosol**: is a simple phenolic compound characterized by a single hydroxyl group at the 4-position of the phenol ring and is prominent in olive oil and wine. Although its low bioavailability it exhibits a wide range of biological activities, including antioxidant, anti-inflammatory and antimicrobial effects and it has been recognized for its cardioprotective and neuroprotective properties [28,30].
- **Oleocanthal**: is a phenolic compound structurally characterized by a phenolic aglycone linked to an aromatic aldehyde group and although it represents approximately 10% of the total polyphenols in olive oil it has notable anti-inflammatory and antioxidant properties [31]. It is also responsible for the distinctive pungent, throat-irritating sensation of extra virgin olive oil, an effect that closely resembles the sensation caused by ibuprofen, with which it shares a similar mechanism of action (i.e., inhibition of cyclooxygenase (COX) enzymes) [32].
- **Oleacein**: is the second most abundant secoiridoid in extra virgin olive oil and it has attracted attention due to its strong antioxidant and anti-inflammatory properties, which are attributed to its structure, similar to that of oleuropein but with a simpler aglycone moiety [33].
- **Elenolic acid**: is a key phenolic compound derived from the hydrolysis of secoiridoids such as oleuropein and ligstroside, and it plays a crucial role in the biosynthesis of various olive-derived bioactives. Structurally, it features a carboxylic acid group and an enolic moiety, which contribute to its chemical reactivity and its role as a building block in the formation of complex phenolics. Although less studied than other olive polyphenols, elenolic acid has shown promising antioxidant and antimicrobial properties [34].

It is remarkable that the biological activity of the olive secoiridoids is related to their chemical structure. This fact is related to the number and position of hydroxyl groups, the presence of glycosylation and the type of aglycone moiety, which influence antioxidant, anti-inflammatory and antimicrobial potency. For example, the ortho-dihydroxy configuration in hydroxytyrosol enhances its radical-scavenging ability [35], while the aldehyde group in oleocanthal contributes to its COX-inhibitory effect, reducing inflammation [36].

Simultaneously with the research on the previously discussed compounds, studies on terpenoids have gained considerable attention for their diverse health benefits, such as anti-inflammatory, antioxidant, and anticancer properties [37]. The primary triterpene found in olive leaves is oleanolic acid, followed by notable concentrations of maslinic acid, and smaller amounts of ursolic acid, erythrodiol, and uvaol [11]. Alongside terpenoids, carotenoids such as β-carotene and lutein, present in both olive fruits and oils, have also shown potential health benefits. They largely bypass absorption in the small intestine and reach the colon, and recent studies suggest that carotenoids may exert prebiotic-like effects by promoting beneficial bacterial shifts, as well as contribute to gut integrity by stabilizing tight junctions and supporting mucosal barrier function [38,39].

Other groups that have gained attention are phytosterols, fatty acids and vitamins, due to their complementary roles in enhancing the nutritional and therapeutic value of olive oil. Phytosterols are structurally similar to cholesterol and have been shown to reduce its intestinal absorption, thereby contributing to cardiovascular health [40]. Fatty acids, particularly oleic acid, represent the predominant lipid fraction in olive oil and are associated with anti-inflammatory, lipid-lowering, and cardioprotective effects [41,42]. Lastly, fat-soluble vitamins such as vitamin E (mainly in the form of α-tocopherol) provide strong antioxidant protection by preventing lipid peroxidation within cell membranes [43]. Collectively, these compounds work synergistically to support the health-promoting properties of olive oil and reinforce its role in preventive nutrition.

### 2.2. Determinants of Olive Oil’s Bioactive Profile

Building on our understanding of the significant health benefits attributed to olive oil’s bioactive compounds, it’s crucial to recognize that their concentration and specific composition are not constant. Instead, these valuable molecules are incredibly dynamic and their presence and potency are shaped by a complex interplay of factors and conditions:

- **Agronomic practices** (e.g., olive variety, climate, soil composition or irrigation): these factors directly impact the biosynthesis of phenolic compounds in the fruit. For example, drought stress can increase the accumulation of certain antioxidants, while specific cultivars like ‘Picual’ (Spain) or ‘Koroneiki’ (Greece) are known for their high polyphenol content [44].
- **Ripeness stage of the olives at harvest**: it affects the profile and abundance of phenolics, as they tend to decrease as olives ripen, with unripe or early-harvested fruits generally containing higher levels of oleuropein and related secoiridoids. The choice of harvest time thus balances yield and bioactive richness [45].
- **Extraction methods**: they play a crucial role in determining both the quantity and quality of recovered bioactive compounds. The choice and optimization of extraction techniques are essential to minimize the degradation of sensitive phenolics while maximizing yield. Conventional methods, such as solvent or mechanical extraction, may not fully recover all valuable compounds or could lead to their alteration. Therefore, newer, greener technologies, like enzyme-assisted or ultrasounds are being developed to enhance selectivity and efficiency, particularly for valorizing olive by-products [46].
- **Thermal treatment**: this is arguably the most critical factor. High temperatures, often encountered during cooking or certain refining processes, can lead to the degradation of heat-sensitive compounds. This includes many polyphenols and fat-soluble vitamins, potentially reducing the oil’s overall nutritional and health-promoting properties [47]. Conversely, some studies suggest that mild thermal processing might, in certain cases, enhance the release or bioavailability of some bound phenolic compounds [48].

Analytical techniques are indispensable for profiling the bioactive composition of olive oil, allowing precise identification, characterization, and quantification of its functional compounds. Techniques such as high-performance liquid chromatography (HPLC), often coupled with diode-array or mass spectrometry detectors (DAD/MS), are commonly employed to characterize the phenolic fraction, while gas chromatography (GC) is used for profiling fatty acids and volatile compounds [49]. The integration of advanced analytical platforms, including metabolomics, is increasingly important to capture the complexity of olive oil’s bioactive matrix and to support innovation in both food quality and health-related research.

## 3. Gut Microbiota

The gut microbiota, a complex and dynamic community of trillions of microorganisms residing primarily in the human colon, plays a fundamental role in maintaining host health. It is involved in a wide array of physiological processes, including nutrient metabolism, immune modulation, intestinal barrier function, and protection against pathogens [20]. A balanced and diverse microbiota composition is essential for homeostasis, while dysbiosis, an imbalance in microbial populations, has been associated with numerous disorders, such as inflammatory bowel disease, obesity, diabetes, cardiovascular disease, and even neurological conditions [2,50,51].

Dietary patterns exert a profound influence on the composition and metabolic activity of the gut microbiota: diets rich in dietary fiber, polyphenols, and fermented foods (like the Mediterranean diet) are strongly associated with increased microbial diversity and enhanced production of SCFAs [20]. Conversely, Western-type diets, characterized by high intake of animal fats, refined sugars, and low fiber, are linked to decreased microbial diversity, increased intestinal permeability, and chronic low-grade inflammation, promoting a state of dysbiosis that predisposes to metabolic and inflammatory diseases [20,52].

### 3.1. Composition and Associated Effects of the Gut Microbiota

In general, the gut microbiota in a healthy individual is mainly composed of bacteria belonging to the phyla Firmicutes and Bacteroidetes, whose balance is often expressed as the Firmicutes/Bacteroidetes ratio, typically ranging from 1 to 3 in healthy adults, while Actinobacteria and Verrucomicrobia also represent significant but smaller proportions. Nevertheless, the relative abundance and diversity of these phyla can vary between individuals due to factors such as age, sex, diet, genetics, environment, and lifestyle habits (e.g., physical activity, sleep patterns, smoking, alcohol consumption, stress) [50,53,54].

These microbial groups play essential roles in maintaining gut homeostasis, including the fermentation of dietary fibers, production of SCFAs, and modulation of the immune system. Within these phyla, genera such as *Lactobacillus* (Firmicutes), *Bacteroides* (Bacteroidetes), *Bifidobacterium* (Actinobacteria), and *Akkermansia* (Verrucomicrobia) have been extensively studied for their beneficial impacts on health and some of the key functions of these beneficial bacteria include:

- **Mucin production and renewal**: for example, *Akkermansia muciniphila* uses mucin as a substrate and, by degrading it, stimulates mucin secretion by goblet cells, reinforcing the mucus layer that lines the intestine [55]. Other genera (e.g., *Lactobacillus* and *Bifidobacterium*) also contribute to maintaining the integrity of the mucosal layer and regulating genes involved in mucin secretion [56].
- **Production of antimicrobial peptides** such as bacteriocins and other peptides, that inhibit the growth of harmful microorganisms, helping to maintain a balanced gut microbiota [57].
- **Colonisation resistance**: for example, *Lactobacillus* competes with pathogenic bacteria for nutrients and adhesion sites on the intestinal mucosa, thereby preventing harmful microbes from establishing themselves in the gut [58].
- **IgA production** due to it influences the immune system by promoting the production of IgA, that plays a crucial role in mucosal immunity and pathogen neutralization [59].
- **Maintenance of the intestinal epithelial cells** as these bacteria contribute to the integrity and function of the intestinal epithelial barrier by supporting cell renewal and tight junctions, which are essential for preventing the translocation of harmful substances [20].
- **Production of SCFAs** such as lactic acid, acetate, propionate, and butyrate, which support gut health by nourishing colonocytes, maintaining intestinal barrier integrity, reducing inflammation, and promoting a balanced gut microbiota [60].
- **Synthesis of proteins**: certain gut bacteria, particularly *Bifidobacterium* and *Lactobacillus*, are capable of synthesizing essential vitamins such as those from the B group (e.g., B12, B9 or folate, B6, B2) and vitamin K, which play key roles in metabolic processes and blood coagulation [61].
- **Modulation of the gut-brain axis** through the production of neurotransmitters like gamma-aminobutyric acid (GABA), serotonin and dopamine, the gut microbiota can influence the central nervous system, affecting mood, behavior and cognitive functions [62].
- **Degradation of xenobiotics**: some microbes can metabolize xenobiotic compounds and pharmaceuticals, potentially altering their toxicity or effectiveness [2].

However, dysbiosis can severely undermine these vital functions, leading to increased intestinal permeability, chronic low-grade inflammation and immune imbalance [63]. Such disruptions have been linked not only to inflammatory bowel diseases but also to metabolic disorders (e.g., obesity, type 2 diabetes), cardiovascular conditions, atopic diseases and even neurodevelopmental or psychiatric disorders like autism spectrum disorder and depression [64,65,66]. Key drivers include excessive antibiotic use, diets low in fiber yet high in fat or sugar, chronic stress and recurrent infections, highlighting that maintaining a diverse and balanced microbiota through prudent antibiotic stewardship, a fiber-rich diet, stress reduction and effective infection control is essential for disease prevention and health promotion [67].

In addition, interactions between conventional foods and commonly used medications may also influence gut microbiota composition. Such interactions can, in some cases, lead to a reduction in beneficial bacterial populations, contributing to dysbiosis and impaired gut function. Notably, several classes of medications (apart from antibiotics), like proton pump inhibitors, non-steroidal anti-inflammatory drugs, and certain antipsychotics, have been associated with significant alterations in microbiota composition, often resulting in decreased microbial diversity. Similarly, some dietary supplements (such as high doses of some minerals), particularly when used excessively or without medical supervision, may negatively impact the balance of commensal bacteria [68,69].

### 3.2. Microbial Influence on Brain and Behavior: The Gut–Brain Connection

An increasingly recognized aspect of gut microbiota research is its bidirectional communication with the central nervous system, known as the “microbiota–gut–brain axis.” This complex network relies on several interconnected systems, including the autonomic and enteric nervous systems, the endocrine system, the hypothalamic–pituitary–adrenal axis, the immune system, and a variety of microbial metabolites [70].

Among these, neurotransmitters and compounds such as essential vitamins, secondary bile acids, amino acids, and SCFAs significantly influence immune signalling pathways, affecting key neurological processes, shaping behavior, cognition, motor activity, and the development or progression of neurodegenerative diseases [71].

In addition, the gut microbiota synthesizes a variety of key neuroactive compounds, including catecholamines (such as noradrenaline, norepinephrine, and dopamine), GABA, serotonin, and a wide range of tryptophan metabolites and precursors. Certain bacterial species can convert neurotransmitter precursors into their bioactive forms; for instance, *Escherichia* are capable of converting glutamate into GABA, while *Lactobacillus* can facilitate the transformation of dietary tryptophan into serotonin [72,73]. These microbiota-derived neurochemicals can interact with host receptors in the intestinal epithelium and vagus nerve terminals, modulating neuronal excitability and influencing signalling pathways that reach the brain. Moreover, changes in the availability of these bioactive molecules have been associated with altered stress responsiveness, sleep regulation, and emotional behaviour [74].

These findings have opened up promising avenues for therapeutic strategies targeting the gut microbiota to prevent or treat neurological and psychiatric disorders: dietary interventions [75], probiotics and prebiotics [76] are currently being explored for their potential to modulate the microbiota–gut–brain axis and support mental and cognitive health. However, more longitudinal and mechanistic studies in humans are needed to fully understand the causal relationships and optimize these interventions.

## 4. Impact of Olive Oil and Its Components in Gut Microbiota

Olive oil contributes to health not only through its high content of monounsaturated fats but also by supplying a diverse array of bioactive molecules. Recent studies have revealed that these compounds engage in two-way communication with the gut microbiota: microbes transform olive oil constituents into new, bioactive metabolites, while those same compounds help reshape the microbial community encouraging the proliferation of beneficial taxa, boosting SCFAs and restraining potentially harmful organisms. Given the central role of the microbiota in overall health, unravelling these reciprocal interactions has become a major focus in nutrition and microbiome research [77].

The lipid-rich matrix of olive oil, particularly its high oleic acid content, not only facilitates the intestinal uptake of phenolic compounds but also independently fosters microbial communities linked to lower inflammatory tone [78]. In turn, the gut microbiota also metabolizes olive polyphenols; for example, hydroxytyrosol is converted into 3,4-dihydroxyphenylacetic acid, a metabolite that supports butyrate-producing bacteria and enhance epithelial barrier function [79].

Olive phenolics, along with other dietary components, fuel microbial fermentation in the gut and may influence both the composition and metabolic activity of the microbial community [1]. Through these interactions, they influence key metabolic pathways enhancing SCFA and secondary bile acid production, modulating bile acid–transforming enzymes, and suppressing microbial trimethylamine formation (the precursor to trimethylamine *N*-oxide, a cardiovascular risk marker) [80,81].

In vitro and in vivo studies have been widely used to investigate the effects of olive oil polyphenols on gut microbiota. In vitro experiments allow for controlled assessment of the direct interactions between polyphenols and specific microbial populations or metabolites, providing mechanistic insights. Meanwhile, in vivo studies, conducted in animal models or humans, offer a more comprehensive understanding of how these compounds influence the complex and dynamic gut ecosystem within a living organism, including effects on host metabolism and immune responses. Combining both approaches enable a thorough evaluation of the impact of olive oil and its bioactive components on gut microbiota composition and function [82]. A selection of representative studies that reflect the relationship between olive derivatives and the gut microbiota is summarized in Table 1 and Table 2.

Considering global perspectives, there is consistent and growing evidence that olive bioactive compounds can effectively modulate the gut microbiota, leading to beneficial effects on gut health in both humans and animal models such as pigs or rodents. These compounds not only may promote a balanced microbial ecosystem but also contribute to enhancing intestinal barrier integrity and reducing inflammation [86,89,90,94]. Moreover, olive bioactives have shown promising therapeutic potential in improving a range of gastrointestinal disorders, including colitis [27,86,94], obesity [89,97], and other forms of intestinal inflammation. Their multifaceted actions, such as antioxidant, anti-inflammatory, and microbiota-modulating effects, highlight their value as natural agents for supporting digestive health and preventing or managing metabolic and inflammatory diseases. Nevertheless, further research is warranted to fully elucidate their mechanisms and optimize their clinical applications.

Phenolic compounds (including polyphenols) have been extensively studied across a wide variety of plants, and it is well established that these compounds promote the growth of beneficial gut bacteria, such as *Lactobacillus* and Bifidobacteriaceae, which are key players in maintaining intestinal health [106]. Overall, only 5–10% of the total polyphenols are absorbed in the small intestine and the rest reach the colon where gut microbiota transforms them into smaller metabolites (e.g., hydroxytyrosol, tyrosol, phenylacetic acids, phenylpropionic acids or benzoic acid derivatives) with better absorption and significant biological effects. These metabolites also display anti-inflammatory properties and enhance the production of SCFAs like butyrate and acetate, which are essential for maintaining gut barrier integrity, modulating inflammation, and supporting energy metabolism [60]. Moreover, polyphenols reduce harmful volatile organic compounds linked to dysbiosis and they improve lipid profiles by increasing monounsaturated (e.g., oleic, palmitoleic) and essential polyunsaturated fatty acids (e.g., omega-3, omega-6) [107].

Although oleuropein is the most extensively studied polyphenol found in olive oil, hydroxytyrosol, a major compound derived from the biotransformation of oleuropein in the gastrointestinal tract, has become the most commonly used compound in both animal and human studies [86,87,89,103,105]. Consequently, many experimental models and clinical trials focus on this molecule to better understand and harness the health benefits associated with olive oil polyphenols, making it a key molecule in translating in vitro findings into practical applications for improving gut health and metabolic function. Moreover, hydroxytyrosol undergoes rapid and extensive metabolism, achieving nearly 99% bioavailability, thanks to its fast intestinal absorbance, which facilitates its systemic circulation and its interaction with target tissues [108].

**Figure 3:**
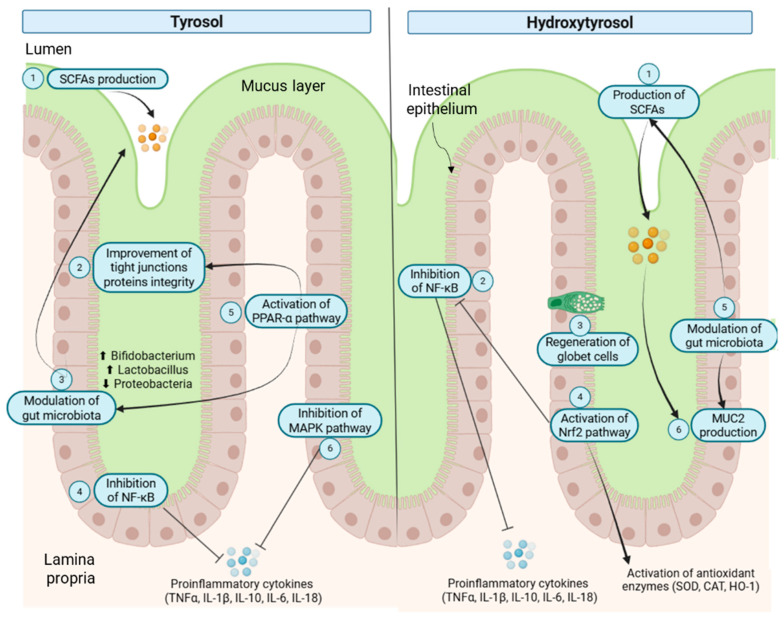
Protective effects of tyrosol and hydroxytyrosol on gut barrier integrity. CAT = Catalase; HO-1 = Heme oxygenase 1; IL = Interleukin; MAPK = Mitogen-activated protein kinase; MUC2 = Mucin 2; NF-κB = Nuclear factor kappa-light-chain-enhancer of activated B cells; Nrf2 = Nuclear factor erythroid 2–related factor 2; PPAR-α = Peroxisome proliferator-activated receptor alpha; SCFAs = Short-chain fatty acids; SOD = Superoxide dismutase; TNF-α = Tumor necrosis factor alpha. Blunt arrows (┴) indicate inhibition.

The success of hydroxytyrosol in improving some gastrointestinal conditions is due to its interaction with the gut microbiota (Figure 3), which plays a pivotal role in modulating the absorption of both the parent compound and its metabolites [109]. This molecule has the ability to activate the nuclear factor erythroid 2–related factor 2 pathway (Nrf2), which helps counteract oxidative stress with the production of antioxidant enzymes such as superoxide dismutase (SOD), catalase (CAT) or heme oxygenase-1 (HO-1) [110]. Additionally, hydroxytyrosol may directly modulate key enzymes and transporters involved in bile acid synthesis, conversion, and excretion, thereby improving bile acid metabolism and overall gut health [111].

In addition, hydroxytyrosol is also capable of reducing the levels of pro-inflammatory cytokines such as TNF-α [112], thereby mitigating intestinal inflammation. It also modulates the synthesis and function of tight junction proteins in the intestinal epithelium, promoting the expression of occludins and upregulating the MUC2 gene, which enhances mucin production and strengthens the intestinal barrier. Moreover, hydroxytyrosol contributes to the differentiation and maintenance of goblet cells, a key epithelial cell type responsible for mucin secretion and crucial for preserving epithelial integrity and immune homeostasis in the gut [87].

Tyrosol is the second most studied olive-derived polyphenol in relation to gut microbiota interactions, although current evidence remains limited compared to hydroxytyrosol. Despite this, tyrosol has shown promising therapeutic potential for various gastrointestinal disorders due to its interaction with the gut barrier (Figure 3); for example, it has been shown that this molecule can reduce inflammation and oxidative stress by inhibiting key signalling pathways such as NF-κB and HIF-1 thereby exhibiting antitumors effects in colorectal cancer [113]. It has also demonstrated significant potential in alleviating colitis symptoms and reducing mucosal damage by enhancing the integrity of the intestinal barrier, promoting the expression of tight junction proteins such as occludins and claudins, and reducing pro-inflammatory cytokine levels [114].

This molecule is also capable of promoting the growth of beneficial bacteria, particularly *Lactobacillaceae* and *Bifidobacteriaceae* and it contributes to the delivery of fatty acids to the cecum and colon, potentially influencing the composition and function of gut microbial communities [115]. In addition, it is able to ameliorate the symptoms of obesity promoting adipose thermogenesis by the modulation of PPAR-α signalling pathway and with the modulation of gut microbiota by decreasing the ratio of Firmicutes to Bacteroidetes and increasing the relative abundance of family Muribaculaceae and of the genus *Blautia* [97].

Apart from hydroxytyrosol and tyrosol, the phenolic compounds oleacein and oleocanthal have been less extensively studied in relation to their impact on the gut microbiota. Currently, evidence remains limited to establish a definitive positive correlation between their presence and microbiota modulation. However, emerging research highlights their potent antioxidant and anti-inflammatory properties, which may play a protective role against intestinal oxidative stress and chronic inflammation, two major factors in the pathogenesis of gut-related disorders [32]. Notably, oleacein treatment has been shown to protect against intestinal mucosal barrier damage in a mouse model of experimental autoimmune encephalomyelitis, reducing colonic levels of inflammatory markers and increasing the abundance of *Akkermansiaceae*, a phylum associated with improved gut health as previously shown [96].

Similarly, oleocanthal intake has been reported to induce both quantitative and qualitative changes in the gut microbiota of mice. Specifically, it promoted the growth of beneficial bacteria such as *Akkermansia muciniphila* and *Lactobacillus*, while decreasing the abundance of pro-inflammatory taxa like *Desulfovibrio*, which is linked to intestinal inflammation and metabolic dysfunction [95]. These multifaceted actions not only support the protective effects of these compounds on gut health but also highlight their promising potential as nutraceuticals in the prevention and management of gastrointestinal and systemic inflammatory conditions. Continued research is warranted to fully elucidate their mechanisms and therapeutic scope.

Research on terpenoids has notably increased because of their broad spectrum of health-promoting properties, such as anti-inflammatory, antioxidant, and anticancer effects [37]. Among the few clinical studies available to date, the following compounds emerge as highly promising candidates for future research in the gut microbiota field:

- Oleanolic acid has been shown in mice to restore microbiota balance, increase SCFAs production, and enhance the expression of intestinal tight junction proteins. These effects are partly mediated by the modulation of key signalling pathways, including downregulation of the NLRP3 inflammasome and activation of the bile acid receptor TGR5, both of which contribute to reduced intestinal inflammation and improved barrier integrity [91].
- Maslinic acid, at both high and low doses, has demonstrated protective effects against Parkinson’s disease and has alleviated damage in alcoholic liver injury models through mechanisms involving the modulation of the gut microbiota [116]. These effects are partly mediated by the modulation of key signalling pathways, including downregulation of the NLRP3 inflammasome and activation of the bile acid receptor TGR5, both of which contribute to reduced intestinal inflammation and improved barrier integrity. Additionally, maslinic acid lowers circulating levels of lipopolysaccharides, leading to a subsequent reduction in pro-inflammatory cytokines, further supporting its anti-inflammatory role in gut homeostasis [90].
- Ursolic acid, has shown potential immunomodulatory and hepatoprotective effects, possibly mediated by modulation of the gut microbiota. Preclinical studies suggest that ursolic acid can increase the abundance of beneficial bacterial genera such as *Odoribacter* and *Parabacteroides*; however, the underlying mechanisms remain poorly understood, and further research is needed to confirm these effects and their physiological relevance in humans [117].

Erythrodiol and uvaol, despite being even less explored, exhibit promising anti-inflammatory properties and may influence gut microbial composition indirectly by reducing oxidative stress and enhancing intestinal epithelial integrity [118,119] and could influence microbial composition indirectly by reducing oxidative stress and improving intestinal epithelial integrity. In summary, the diverse bioactive compounds in olive oil exhibit significant potential in modulating gut microbiota composition and function, thereby contributing to improved gut health and systemic metabolic regulation. Their multifaceted effects, ranging from enhancing beneficial bacterial populations and SCFAs production to strengthening intestinal barrier integrity and reducing inflammation, highlight their importance as natural agents in preventing and managing gastrointestinal and metabolic disorders. Further well-designed clinical studies are essential to fully elucidate their mechanisms, optimize dosages, and translate these findings into effective nutraceutical and therapeutic interventions. The continuing exploration of olive oil bioactives within the gut microbiome landscape holds great promise for advancing personalized nutrition and improving human health outcomes.

## 5. Current Challenges and Future Directions

Understanding the interactions between olive-derived bioactive compounds and the gut microbiota has emerged as a promising field with important implications for human health. Numerous in vitro and in vivo studies have demonstrated that phenolic compounds, secoiridoids, and triterpenes present in olives and their by-products can modulate the composition and functionality of the intestinal microbiota, contributing to improved metabolic, inflammatory, and intestinal barrier outcomes. However, despite growing evidence supporting their beneficial effects, much remains to be clarified about the specific mechanisms involved, the variability of individual responses, and the translatability of preclinical findings to human physiology, limitations that represent a typical caveat in gut microbiome research rather than an issue unique to olive-derived interventions [120].

### 5.1. Harnessing the Collective Power of Olive’s Bioactives in Gut Health

Future research should prioritize the investigation of the potential synergistic effects arising from the combination of the diverse bioactive compounds present in olive oil. While individual compounds such as hydroxytyrosol, oleuropein, maslinic acid, and oleanolic acid have been extensively studied in isolation, it is increasingly evident that their combined action may produce additive or even synergistic biological effects that surpass those of the single constituents. Exploring these interactions at molecular, cellular, and systemic levels could provide critical insights into the multifaceted mechanisms underlying the health benefits attributed to olive oil consumption. Advanced experimental designs integrating metabolomics, microbiome profiling, and systems biology approaches will be indispensable to elucidate how these compounds collectively modulate gut microbiota composition, microbial metabolite production, and host physiological responses. Such research is essential to optimize the therapeutic applications of olive oil bioactives, potentially leading to the development of novel nutraceutical formulations or dietary interventions that exploit these synergistic properties for enhanced efficacy in preventing and managing metabolic, inflammatory, and neurodegenerative diseases.

### 5.2. Challenges in Translating Animal Model Findings to Human Microbiota Research

Although rodent models provide valuable insights into diet–microbiota interactions, mainly due to the ability to tightly control variables such as genetics, diet, and microbial composition, caution is needed when extrapolating these results to humans. Laboratory animals are usually genetically uniform and kept in highly controlled environments, ignoring many external and host-related factors that contribute to the complexity and variability observed in the human gut microbiome, while human populations exhibit far greater genetic, microbial, and phenotypic diversity, which animal models cannot fully replicate. Furthermore, differences in gut physiology, immune responses, microbiota composition, and even metabolism between rodents and humans may lead to divergent outcomes in response to the same compounds. Therefore, future studies should prioritize well-designed clinical trials and include diverse human populations to validate preclinical findings and better understand individual variability in gut microbiota modulation by olive-derived bioactives.

### 5.3. Complexities and Challenges in Studying Olive Impact on the Microbiota-Gut-Brain Axis

While preliminary evidence suggests that olive oil and its bioactive compounds may modulate the microbiota–gut–brain axis, the extent and biological relevance of this interaction remain to be fully elucidated. That problem remains in the complexity of standardizing the olive oil composition (especially its minor bioactive compounds) as well as the inherent variability of gut microbiota and the lack of consensus on what constitutes a “healthy” microbial profile. Most available data have been derived from animal studies, highlighting the urgent need for well-designed, long-term human trials that consider dietary context, intervention duration, and individual variability. Moreover, future research should aim to unravel the specific molecular mechanisms of neuromodulation mediated by microbiota-derived compounds and assess their clinical implications in disorders such as depression, cognitive decline, or neurodegenerative diseases. Advancing this field holds great promise not only for scientific understanding but also for the development of dietary interventions with significant public health impact.

### 5.4. Limitation of Epidemiological Studies

Epidemiological studies often help to identify associations between exposures and health outcomes in humans, reflecting real-world scenarios. However, these studies come with inherent limitations, including the difficulty of objectively measuring dietary adherence, an issue that also affects randomized controlled trials, and the challenge of establishing true control groups. Most research tends to compare healthy individuals with patients, often overlooking the shared environmental and dietary influences on the microbiota. Incorporating analyses of household members or siblings could help clarify these environmental effects [1].

Additionally, confounding factors related to diet, including inconsistent adherence to dietary protocols, the vast diversity of food components consumed, and the impact of diet-induced changes on host metabolism independent of the microbiota, make it challenging to establish direct cause-effect relationships between specific dietary compounds and health outcomes. To address this, Vujkovic-Cvijin and colleagues have proposed a comprehensive set of host variables [121], ranging from physiological to lifestyle and dietary factors, that should be controlled for in human microbiota studies to improve the reliability and reproducibility of findings linked to disease.

### 5.5. Study of Microbial Metabolites Beyond SCFAs

Alongside the well-documented influence of olive oil bioactive compounds on SCFAs production, there is growing recognition that these compounds may also modulate a broader array of microbial metabolites with important physiological implications. These include bile acids, which play a pivotal role in lipid digestion and metabolic signalling; neurotransmitters such as γ-aminobutyric acid and serotonin precursors, which are central to the communication between the gut and the brain via the gut-brain axis; and various phenolic metabolites that result from microbial transformation of dietary polyphenols. These metabolites can have systemic effects beyond the gut, potentially influencing neurological function, immune responses, and metabolic regulation.

## 6. Concluding Remarks

Olive oil-derived polyphenols, secoiridoids and triterpenes interact bidirectionally with the gut microbiota to shape the production and balance of diverse microbial metabolites, beyond SCFAs to include secondary bile acids, neurotransmitters and phenolic derivatives, thereby underpinning their metabolic, anti-inflammatory and neuroprotective benefits. Translating these insights into dietary recommendations and functional products will require multidisciplinary, integrative studies that combine clinical trials with advanced multi-omics and systems biology approaches. By deepening our mechanistic understanding and standardizing olive oil compositions, we could fully unlock the therapeutic potential of olive bioactives for metabolic, inflammatory and gut brain axis related disorders.

## Figures and Tables

**Table 1:** Representative in vitro and in vivo animal studies on the effects of olive-derived bioactive compounds on gut microbiota composition and function.

Animal Studies						
Title	Study Type	Olive Matrix	Compound	Dosage and Time	Microbiota Changes	Main Findings
[83]	In vivo	Olive leaves	OLE	2.7 g of OLE/kg of diet during 1 week	↑ Clostridiales, *Paraprevotella*, *Anaerotruncus*, *Oscillibacter*, *Sporobacter* ↓ *Alistipes*, *Morganella*	OLE significantly modulated the gut microbiota and plasma metabolomic profiles, supporting its potential protective role in colorectal cancer progression
[84]	In vivo	Olive leaves	OLE	1 g/kg and 2 g/kg feed for 31 days	↑ *Lactobacillus* ↓ *E. coli*, *Clostridium* spp.	↑ body weight, ↑ carcass and breast yield, ↓ abdominal fat; improved lipid profile (↓ cholesterol, TG, VLDL); enhanced immunity and antioxidant markers
[85]	In vivo	Olive mill wasterwater	HT, P	74 ppm and 225 ppm of polyphenols/pig/day for 85 days	↑ *Eubacterium*, *Treponema* ↓ *Fusobacterium*, *Bacteroides helcogenes*, *Corynebacterium urealiticum*	Positive effect on gut health and microbiota composition; improvement of intestinal morphology and potential to improve animal growth and health
[86]	In vivo	Olive oil	HT	10 and 50 mg/kg of HT	↑ Lachnospiraceae, Muribaculaceae, *Colidextribacter* ↓ Bacteroidaceae, Desulfovibrionaceae	HT promoted intestinal barrier regeneration, modulated gut microbiota composition and restored SCFAs production in colitic mice
[87]	In vivo	-	HT	500 mg/kg HT for 28 days	↑ *Clostridium*, *Intestinibacter* ↓ *Lactobacillus*, *Streptococcus*	HT Improves intestinal morphology (↑ villus height, ↑ villus height/crypt depth ratio) and attenuates intestinal inflammation enhancing antioxidant capacity
[88]	In vivo	-	HT	60 mg/kg of HT after 7 days of induction of colitis with DSS	*↑* Bacteroidota, Bacteroidota, Firmicutes *↓ Staphylococcus*, *Aerococcus*, *Alistipes*	HT alleviated the DSS-induced colitis and enhanced antioxidant and anti-inflammatory responses
[89]	In vivo	-	HT	50 mg/kg/day of HT	*↓* Ruminococcaceae, Proteobacteria, Christensenellaceae, *Ferribacter*; *↑ Parabacteroides goldsteinii*, *Lactobacillus johnsonii*, *Rikenella*	HT modulated gut microbiota composition, reversed dysbiosis and alleviated inflammation in High-Fat-Diet-Induced mice
[90]	In vivo	-	MA	50 and 100 mg kg^−1^ of MA during 11 days	*↑* Bacteroidetes, *Parabacteroides* *↓* Proteobacteria, *Enterococcus*, *Escherichia-Shigella*	MA alleviates alcoholic liver injury, intestinal barrier damage and inhibits liver inflammation
[91]	In vivo	-	OA	100 mg/kg of OA per day	*↑* Firmicutes, *Oscillibacter*, *Ruminiclostridium*; *↓* Actinobacteria	OA changed the composition of mice gut microbiota and improved the intestinal immune system
[92]	In vitro	Olive leaves	PC, OLE	Not specified	*↑* Coriobacteriaceae (*Collinsella* sp.), Eggerthellaceae (*Eggerthella* sp.) *↓* Bacteroidota	↓ oleuropein and ↑ hydroxytyrosol and phenolic metabolites; significant changes in amino acids and fatty acids
[93]	In vivo In vitro	Olive leaves	P	800 g leaves/sheep/day; 30 days	*↑ Catenisphera*, *Mogibacterium*	Increase in milk MUFA, PUFA (n-3 and n-6), decrease in SFA and improved milk fatty acid profile and health indexes
[94]	In vivo	-	Tyr	20 mg Tyr/kg/day for 7 days	↑ *Lactobacillus*, *Bifidobacterium*, *Akkermansia* ↓ *Proteus*	Tyr alleviated colitis morbidity and mucosal injury and improved colonic barrier integrity
[95]	Ex vivo	Extra virgin olive oil	OC	10 mg of OC	*↑ Monoglobus pectinilyticus*, Lachnospiraceae *↓ Prevotella denticola*, *Bacteroides caecimuris*	Positive potential of oleocanthal as a nutraceutical
[96]	In vitro/Ex vivo	Olive leaves	OLEA	Not specified	*↑* Akkermansiaceae	Oleacein regulate intestinal oxidative stress, inflammation and permeability in mice.
[97]	In vivo	Extra virgin olive oil	Tyr	0.2% (wt/wt) for 16 weeks	*↑* Muribaculaceae, *Blautia*, Lachnospiraceae *↓* Firmicutes/Bacteroidetes	Tyrosol consumption attenuates obesity and related symptoms in HFD-fed mice probably via the modulation of PPARα-thermogenesis and gut microbiota
[98]	In vivo	-	UA	25, 50 and 100 mg/kg (bw)/day UA	*↑* Firmicutes/Bacteroidetes	UA alleviates the symptoms of type 1 diabetes mellitus in rats and it modulates the intestinal microflora composition

HT: hydroxytyrosol, MA: maslinic acid, P: polyphenols, PC: phenolic compounds, OA: oleanolic acid, OC: oleocanthal, OLEA: oleacein, OLE: oleuropein, Tyr: tyrosol, UA: ursolic acid. ↑ indicates increased abundance; ↓ indicates decreased abundance.

**Table 2:** Representative in vitro and in vivo human studies on the effects of olive-derived bioactive compounds on gut microbiota composition and function.

Title	Study Type	Olive Matrix	Compound	Dosage and Time	Microbiota Changes	Other Findings
[99]	In vitro	Olive pomace	P	Not specified	↑ Bifidobacteriaceae, Lactobacillales	↑ low organic fatty acids; ↓ detrimental volatile organic compounds (e.g., skatole)
[100]	In vitro	Olive leaves	OLE	Not specified	↓ α-diversity after 24 h	Biotransformation of OLE was via deglycosylation, hydrolysis, ring cleavage, demethylation
[101]	In vitro	Extra virgin olive oil	-	40 mL/day of Extra virgin olive oil		Reduced inflammation and improved gut microbiota health (increase in SCFA levels)
[102]	In vivo	Virgin olive oil	PC	80 mg/kg–500 mg/kg of PC	↑ IgA-coated bacteria	Increase in systemic inflammation markers contrasts with known anti-inflammatory effects of olive oil phenolics, suggesting a complex dose-dependent response
[103]	In vivo	Olive pomace	HT	Not specified	*↑ Bifidobacteria*; *↓ Lactobacilli*, *Ruminococcus*	Sex-dependent variation in metabolites and bacteria abundances and trend to ↓ oxidized LDL cholesterol
[104]	In vitro	Olive pomace	P	-	*↑ Prevotella*, *Bacteroides* and stable ratio of Firmicutes/Bacteroidetes	Production of SCFAs (acetate, butyrate, propionate) and development of a mucin-adhesion inhibition ability against pathogens
[105]	In vitro	Olive leaves	HT, Ty, OLE, vanillin	Not specified	*↑ Bifidobacterium*, *Clostridium* *↓* Firmicutes:Bacteroidetes *ratio*	↑ SCFAs (acetate, butyrate, propionate)

HT: hydroxytyrosol, MA: maslinic acid, P: polyphenols, PC: phenolic compounds, OA: oleanolic acid, OC: oleocanthal, OLEA: oleacein, OLE: oleuropein, Tyr: tyrosol, UA: ursolic acid. ↑ indicates increased abundance; ↓ indicates decreased abundance.

## Data Availability

The original contributions presented in the study are included in the article, further inquiries can be directed to the corresponding author.
